# The case of the unwanted crystal: a case of pediatric pulmonary *Actinomyces Odonolyticus*

**DOI:** 10.1002/ccr3.1555

**Published:** 2018-05-10

**Authors:** Ashley Gray, Paul Do

**Affiliations:** ^1^ Department of Pediatrics Fresno Graduate Medical Education University of California San Francisco 155 North Fresno Street Fresno California 93701‐2302 USA

**Keywords:** Actinomyces odontolyticus, pulmonary actinomyces, sulfur crystal

## Abstract

This case report is one of the only known cases of Actinomyces odontolyticus causing thoracic disease in an immunocompetent pediatric patient. This case also exemplifies how bronchoscopy was able to remove the nidus of infection and prevent the potential for significant morbidity associated with a lobectomy.

*Actinomyces odontolyticus* is an insidious, Gram‐positive, anaerobic bacilli, is a typical flora of the buccal mucosa, and is known to cause chronic cervicofacial infections 1. *A. odontolyticus* rarely causes disease in immunocompetent individuals, especially children. Thoracic disease is thought to be due to aspiration of oropharyngeal secretions leading to pneumonia or abscess 2, 3. Histology is the only definitive diagnosis, and many patients require surgical intervention 4.

An 11‐year‐old female with recurrent pneumonia presented for chronic cough for 2 years. She was previously treated with four courses of antibiotics for a left lung infiltrate without improvement. Physical examination was remarkable for cough and grade two tonsillar hypertrophy. She was initially treated with amoxicillin/clavulanic acid for a presumed protracted bacterial pneumonia. Laboratories including a sweat chloride test, tuberculosis screen, and immunoglobulin levels were all within normal limits. Chest computed tomography 1 month later showed left lower lobe bronchiectasis (Fig. 1). Brush biopsies from her initial bronchoscopy revealed a left lower lobe mucoid mass which grew *A. odontolyticus*. She was started her on penicillin V and referred to cardiothoracic surgery who recommended a left lobectomy. We opted for medical management and on repeat bronchoscopy 6 months later a large amber‐colored crystal was retrieved from the left lower lobe which was identified as *A. odontolytic*us by pathology (Fig. 2). She will be referred to otolaryngology for tonsillectomy as a potential origin of her infection. Repeat bronchoscopy cultures were negative for *Actinomyces* 1 year later.

**Figure 1:**
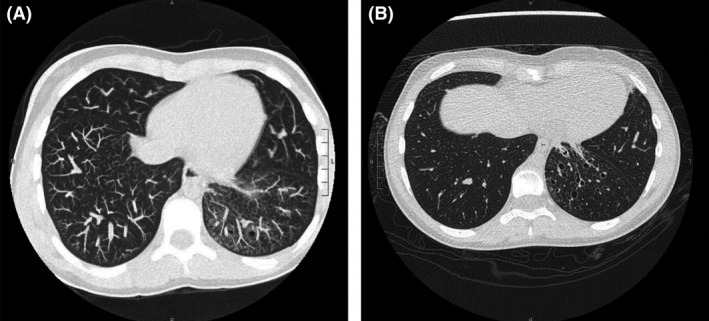
Computed topography of the chest. (A) Initial imaging remarkable for cystic bronchiectasis with scattered reticular and ground‐glass nodular opacities throughout left lower lobe. (B) Six months later, the lung parenchyma is essentially unchanged from previous examination.

**Figure 2:**
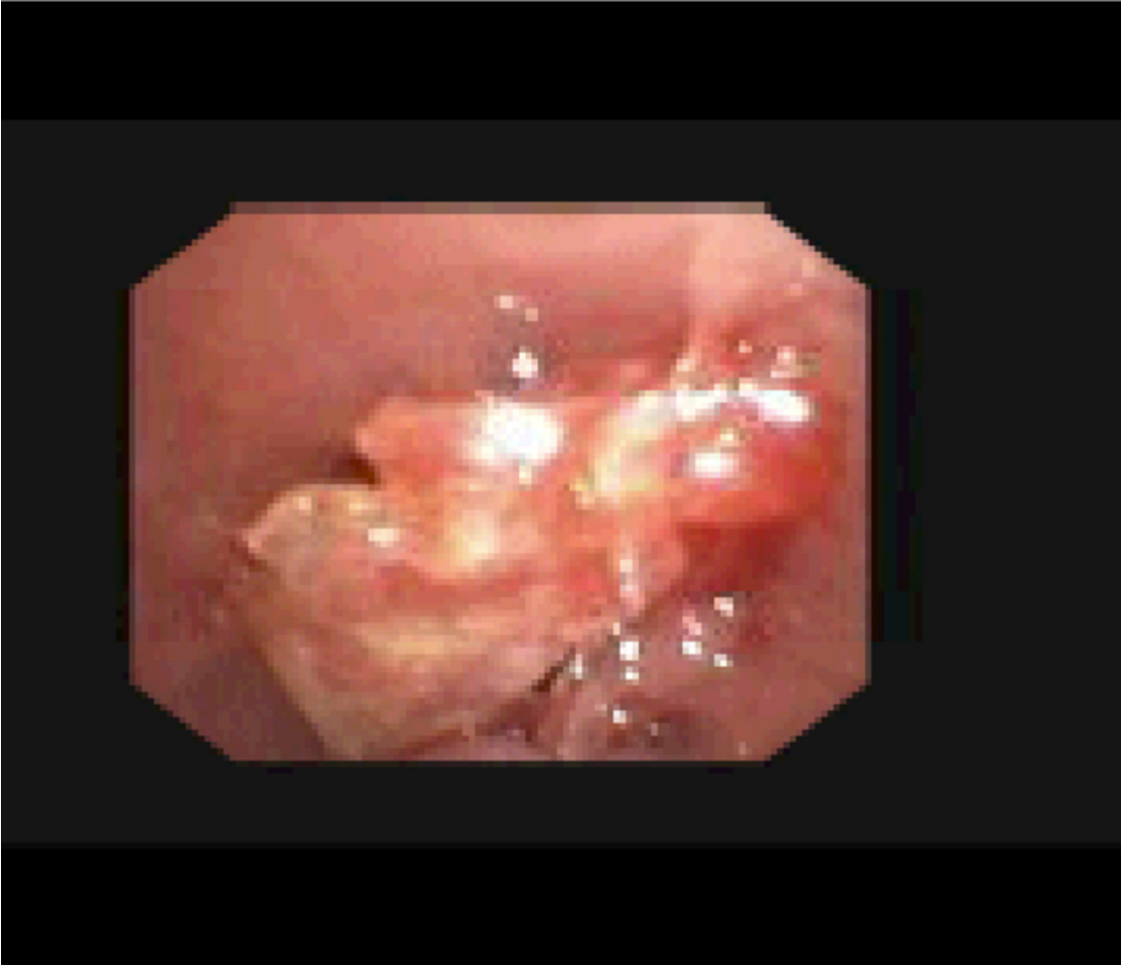
Left lower lobe of lung with an impacted crystal‐like mass with surrounding erythema and inflammatory changes noted on bronchoscopy.

This is the only known reported case of *Actinomyces odontolyticus* causing thoracic disease in an immunocompetent child. This case exemplifies the importance of direct bronchoscopic removal of the *Actinomyces* crystal that prevented a prolonged antibiotic course and potential lobectomy, which patients often require to prevent further progression of the disease.

## Authorship

AG: completed submission and revisions of the manuscript as the primary writer of the manuscript. PD: provided revisions to manuscripts and assistance with submission of the manuscript.

## Conflict of Interest

None declared.
